# The Interplay between Adeno-Associated Virus and Its Helper Viruses

**DOI:** 10.3390/v12060662

**Published:** 2020-06-19

**Authors:** Anita F. Meier, Cornel Fraefel, Michael Seyffert

**Affiliations:** Institute of Virology, University of Zurich, CH-8057 Zurich, Switzerland; anita.meier@uzh.ch (A.F.M.); cornel.fraefel@uzh.ch (C.F.)

**Keywords:** adeno-associated virus, herpes simplex virus, adenovirus, viral coinfections, helper virus, viral vectors

## Abstract

The adeno-associated virus (AAV) is a small, nonpathogenic parvovirus, which depends on helper factors to replicate. Those helper factors can be provided by coinfecting helper viruses such as adenoviruses, herpesviruses, or papillomaviruses. We review the basic biology of AAV and its most-studied helper viruses, adenovirus type 5 (AdV5) and herpes simplex virus type 1 (HSV-1). We further outline the direct and indirect interactions of AAV with those and additional helper viruses.

## 1. Introduction

Arguably the most prominent and popular aspect of adeno-associated virus (AAV) is its use in gene therapy with over 200 currently ongoing clinical trials (http://www.abedia.com/wi-ley/vectors.php). Nevertheless, in this review, we focus on another, equally fascinating aspect: the interplay between AAV, its helper viruses, and the impact on the coinfected host cell. We first outline the biology of AAV, herpes simplex virus type 1 (HSV-1), and adenoviruses (AdVs), followed by the exploration of the interactions between AAV and its helper viruses. The role of AAV in gene therapy is briefly discussed at the end.

### 1.1. Adeno-Associated Virus

Adeno-associated virus (AAV) was discovered in the 1960s as a contaminant of a simian adenovirus type 15 preparation [1,2]. Since then, AAV has been successfully developed into a clinically used viral vector, with Glybera (alipogene tiparvovec) as the first-ever approved gene therapy treatment.

AAV belongs to the *Dependoparvovirus* genus within the *Parvoviridae* family. At least 12 naturally occurring serotypes have been discovered, which vary in their tissue tropism [3,4]. This particular aspect is used for targeting AAV gene-therapy vectors to the site of interest. AAV infects a wide range of animals, including humans, and is found worldwide with a seroprevalence in the human population ranging from around 15% to over 90%, depending on the AAV serotype as well as the cohort studied (e.g., 96.6% for AAV2, 82% for AAV8, and 40.2% for AAV5 in a Chinese cohort study) [5,6,7]. Infection with AAV is asymptomatic and can remain lifelong. As the genus name suggests, AAV can only replicate in the presence of helper factors, which are provided by coinfections by helper viruses from the herpesvirus family (e.g., HSV-1 and human cytomegalovirus, HCMV), adenoviruses (e.g., AdV5), and papillomaviruses (e.g., human papillomavirus type 16, HPV-16), as well as other viruses such as baculovirus and human bocavirus 1 [1,8,9,10,11,12]. Interestingly, AAV replication can also be induced by treating AAV-infected cells with physical or chemical carcinogens and is, thus, not inherently dependent on viral coinfections but rather on a dramatic change in the cellular environment [13,14,15,16]. Without helper factors, AAV delivers its genome into the host cell where most copies are cleared after a short time, while some of the AAV genomes persist long-term [17]. Long-term persistence is believed to predominantly occur in an episomal, circular form [18]. Latently persisting AAV reactivates upon coinfection with a helper virus, which results in the production of progeny virus. Further details about AAV biology are described in Section 1.2 below. Our main focus in this review lies on AAV2 since it is the best-studied of all AAV serotypes.

### 1.2. AAV Biology

AAV is a small, nonenveloped virus of 25 nm in diameter with a single-stranded DNA (ssDNA) genome of 4.7 kb (reviewed in [19]). Its capsid is of icosahedral shape with T = 1 symmetry and is composed of 3 viral proteins, VP1, VP2, and VP3, which are present in approximately a 1:1:10 ratio, with VP3 as the most abundant protein [20,21,22]. Although AAV has a very limited genome size (4.7 kb), multiple open reading frames (ORFs) and alternative splicing expand its coding capacity. The AAV genome consists of two coding regions termed *rep* and *cap*, with three ORFs encoding eight viral proteins regulated by three different promoters [19,23]. These promoters are termed *p5*, *p19,* and *p40* according to their map position on the genome (position 0.06, 0.19, and 0.385, respectively) [24,25,26,27]. The mRNAs resulting from transcription promoted by *p5* and *p19* give rise to the regulatory (Rep) proteins [28]. Full-length *p5* mRNA is translated into Rep78 and its spliced mRNA into Rep68. Full-length *p19* mRNA translation results in Rep52 and its spliced mRNA in Rep40. From the *p40* promoter, two spliced mRNAs give rise to the three capsid proteins (VP1, VP2, VP3) as well as the assembly-activating protein (AAP) by using four different start codons. VP1 is translated from the minor spliced mRNA [29]. VP2, VP3, and AAP are translated from the major spliced mRNA, with VP3 having a conventional AUG start codon, whereas VP2 has a weak ACG start codon [29,30]. AAP is translated from yet another weak start codon (CTG) in a different reading frame. The AAV coding sequence is flanked by two noncoding sequences, the inverted terminal repeat (ITR) sequences [31]. Although noncoding, the ITRs are an essential part of the AAV genome. ITRs self-assemble into a T-shaped double-hairpin structure and provide self-priming activity for DNA replication as well as the packaging signal [32,33]. The ITRs are the only *cis*-acting elements required for genome packaging and, therefore, the only AAV-derived sequences required for recombinant AAV (rAAV) vectors [34]. Furthermore, ITR sequences can mediate the integration of AAV into target sequences within the host genome or plasmids (reviewed in [35]). There is no preference of polarity for packaging, meaning that positive and negative strand genomes are packaged equally well and show the same transduction efficiency [36,37,38].

**Figure 1:**
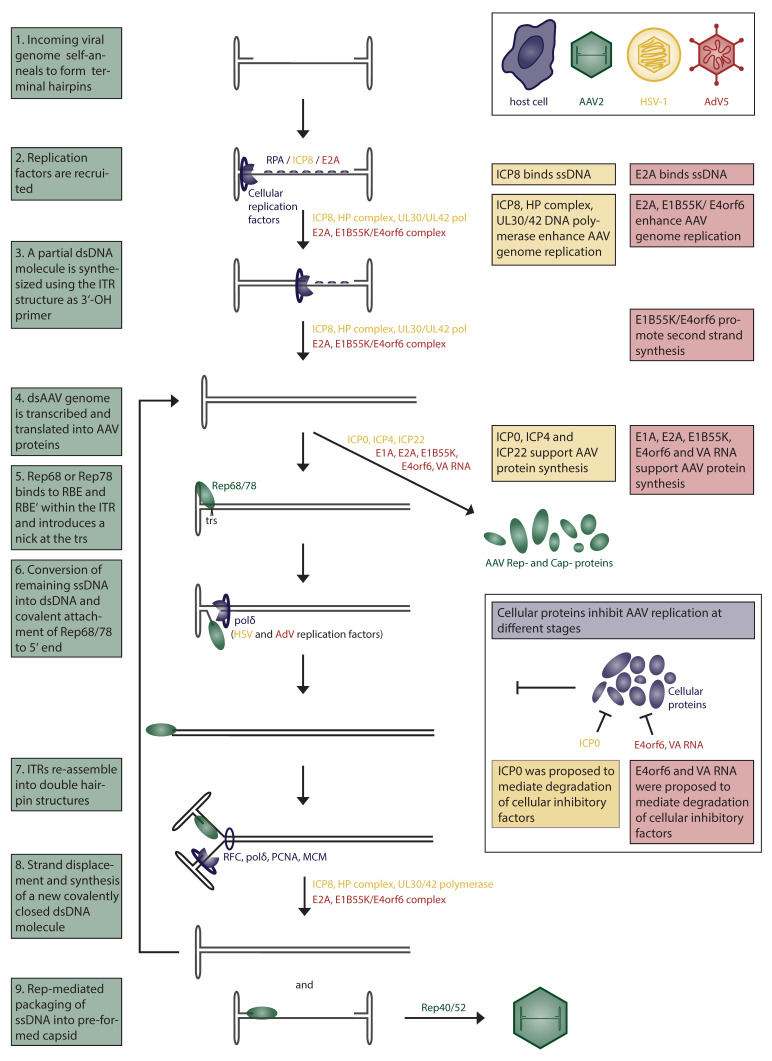
Boxed texts on the left (green) describe each step during AAV replication. The role of HSV-1 (yellow) or AdV5 (red) helper factors and interfering cellular factors (purple) are indicated in the figure and described on the right. AAV Rep-proteins are depicted in green. HSV-1 and AdV5 helper factors in Step 6 are unclear and, therefore, put in brackets.

AAV enters the cell upon attachment to a primary receptor, followed by endocytosis (reviewed in [39]). Different serotypes were found to bind to different cell receptors. AAV2, AAV3, and AAV6 attach to heparan sulfate proteoglycan (HSPG), AAV1, AAV4, AAV3, and AAV6 to sialic acids and AAV9 to N-linked galactose (reviewed in [39]). A role of coreceptors had been suggested, but later studies failed to confirm their importance in AAV attachment and uptake [40,41,42,43,44]. Interestingly, AAV2 isolated directly from human tissues did not bind to HSPG, suggesting cell culture adaptation of lab strains and alternative AAV2 receptors in vivo [45]. An unbiased genetic screen identified a transmembrane protein, termed AAV receptor (AAVR), as an essential factor for AAV transduction for multiple serotypes [44]. Revisiting that same data set, as well as a follow-up study, identified GPR108 as another essential factor for AAV entry, which was proposed to act downstream in the same pathway as AAVR [46]. AAVR is present on the cell membrane and is transported to the trans-Golgi network (TGN) in a retrograde endosomal fashion. Different endocytic pathways were suggested to play a role during entry, but the clathrin-independent carrier (CLIC)/GPI-anchored protein-enriched early endosomal compartments (GEEC) pathway was shown to be the major endocytic route of infection [47,48]. The exact mechanism of AAV trafficking from early endosomes to the cytoplasm remains to be fully understood. One model suggests that AAV is transported from early endosomes to the TGN/Golgi apparatus and from there escapes into the cytoplasm from where it enters the nucleus [49]. Transport of AAV2 to the Golgi apparatus was indeed found to be necessary for transduction, supporting the idea of retrograde endosomal transport. Retrograde transport through the endosomal system is a highly regulated and selective process, which allows the cell to retrieve and recycle proteins and lipids from the plasma membrane and enable their localization to the Golgi (TGN and Golgi apparatus) as well as to the endoplasmic reticulum (ER). Low pH in endosomal compartments and possibly the activity of proteases trigger a conformational change in the AAV capsid, exposing the N-terminal domain of the largest capsid protein VP1 [50]. This so-called VP1 unique region (VP1u) contains a phospholipase A2 domain (PLA2) as well as a nuclear localization signal, permitting the escape into the cytoplasm as well as allowing nuclear import [51,52,53,54]. Once located within the nucleus, AAV2 was found to accumulate in the nucleoli in an intact infectious state [55]. The mechanism of AAV uncoating is largely unknown and seems to be a limiting step during AAV transduction [56,57]. Both wildtype (wt) AAV as well as rAAV can integrate into the host genome [58]. In the presence of Rep78, AAV is able to integrate into the host genome, preferentially at a locus on chromosome 19 termed *AAVS1* [58,59]. Although AAV genome integration can occur, several studies have shown that the frequency of integration within *AAVS1* and outside is low, with 0.1–0.5% of added infectious particles integrating [60,61]. Genomes of AAV-derived viral vectors were found to circularize over time in cell culture and in vivo, supporting the notion of a predominantly circular episomal state during latency [18]. Binding of Rep78 and Rep68 to a specific sequence within the *p5* promoter, the Rep binding element (RBE) suppresses transcription during AAV latency, whereas binding to the RBE sequence within the ITR activates transcription [62]. Upon helper virus coinfection, AAV transitions into its lytic stage, which leads to genome amplification and packaging. AAV genome replication has been fully reconstituted in vitro and will be further described in detail (Figure 1). The role of helper viruses during AAV replication is discussed below in Section 2 of this review. The minimal set of factors necessary for AAV genome replication in vitro are the cellular replication factor C (RFC), polymerase δ, proliferating cell nuclear antigen (PCNA), minichromosome maintenance (MCM) complex, as well as the AAV Rep proteins Rep78 or Rep68 [63,64]. AAV DNA replication in crude cell extracts additionally requires an ssDNA binding protein, such as the cellular replication protein A (RPA) [65]. The two large Rep proteins possess helicase as well as endonuclease activities, which are both essential for AAV DNA replication. AAV replicates by a strand-displacement mechanism using the ITR as a primer to initiate second-strand synthesis resulting in a covalently closed duplex structure [66]. To resolve the duplex structure, the *terminal resolution site* (*trs*) within the ITR sequence is nicked by Rep78 or Rep68, and the remaining part is converted into dsDNA [67]. AAV capsid assembly is supported by the viral assembly-activating protein (AAP), and its requirement ranges broadly across serotypes [68]. Newly synthesized AAV DNA is packaged into preassembled capsids, which have an 8.5-Å diameter pore through which the genome is taken up [69]. This process requires the helicase activity of the small Rep proteins, Rep40 and Rep52, which function as a motor for loading the ssDNA genome into capsids [70]. A large Rep protein, covalently attached to newly formed AAV genomes, might serve as a packaging signal as it was shown that *p5*-containing (and ITR-lacking) sequences, which are bound by Rep, can be packaged into AAV-capsids [71].

## 2. Helper Viruses and AAV

AAV is able to infect host cells in the absence or presence of helper factors, but replication can only occur if the cellular environment is altered dramatically. Although coinfection with specific helper viruses has been shown to induce and support AAV replication, it is not an absolute requirement, as shown by in vitro AAV replication studies as well as upon treatment of AAV-infected cells with carcinogens [13,14,15,16,63,64]. The most studied AAV helper virus is AdV5, presumably because AAV was discovered in an AdV preparation [1,2]. Another well-studied helper virus is HSV-1. In this review, we describe the interplay of these two helper viruses with AAV in detail (Figure 1). Although less well studied, we further delve into the interaction of AAV with some additional helper viruses. 

Many other members of the herpesvirus family have been shown to support productive AAV replication, such as HCMV, herpes simplex virus type 2 (HSV-2), varicella zoster virus (VZV), and human herpesvirus 6 (HHV-6) [9,72,73,74]. In addition, it was shown that an autonomous parvovirus, the human bocavirus 1, can act as a helper virus during AAV replication [12]. Furthermore, the production of rAAV was established in a baculovirus system lacking any HSV or AdV helper genes [75].

### 2.1. Herpesviruses

*Herpesviridae* is a large family of dsDNA viruses, which comprise 8 human pathogens and many additional viruses infecting other species. Members of the *Alphaherpesvirinae* subfamily within the *Simplexvirus* genus include HSV-1 and HSV-2, which cause oropharyngeal or genital mucosal lesions, respectively. Less frequently, they cause encephalitis and meningitis. In this review, we will focus on the biology of HSV-1 since it is the best-studied AAV helper virus within the *Herpesviridae* family.

#### 2.1.1. HSV-1 Biology

HSV-1 is a large enveloped virus of about 150–200 nm in diameter (and about 225 nm, if the length of envelope spikes is considered) and is structurally organized into three different layers [76,77]. The outermost layer, the lipid bilayer envelope, is acquired during viral egress and contains at least 12 different viral glycoproteins, some of which mediate attachment and entry into the host cell during infection. Within the tegument, the layer below the envelope and above the capsid, over 20 virus-encoded proteins were identified [66]. The innermost compartment, the highly pressurized icosahedral capsid, which is about 125 nm in diameter, tightly packs the virus genome [78,79]. HSV-1 has a linear 152 kb long dsDNA genome, which encodes over 80 proteins. The HSV-1 genome can be divided into two parts, a long and a short segment, termed *unique long* (*U_L_*) and *unique short* (*U_S_*). Both segments are flanked by inverted repeat regions. Those two segments rearrange upon DNA replication but only in the so-called *a* sequence within their repeat regions, leaving the unique sequences unmodified while their orientation to each other can be inverted [80,81]. Based on their location within the genome, HSV-1 genes were termed either *UL* or *US*, followed by a number. The gene encoding ICP8, for example, was named *UL29* and is located within the *U_L_* region and has the number 29. HSV-1 genes are further categorized according to their timing of expression during an infection. First, immediate-early genes (IE or *α*) are expressed, then early (E or *β*), and finally, late genes (L or *γ*) [82]. Late genes are further divided into leaky late (*γ1*) and true late (*γ2*) genes, with leaky late gene expression being independent and true late genes being dependent on viral DNA-replication. Although the classification of HSV-1 genes into IE, E, and L genes is convenient, gene expression takes place in a gradual process rather than in distinct stages. Due to the large number of HSV-1 genes, we will only highlight the ones relevant for a basic understanding of HSV-1 biology and relevant in terms of AAV coinfections.

HSV-1 infects epithelial cells from a wide variety of species and tissues, including some of the common lab cell lines such as HeLa cells (human cervix cancer), Hek293 cells (human epithelial kidney), and Vero cells (green monkey kidney). In natural infections, HSV-1 primarily infects epithelial cells of the oral cavity, causing cold sores. After an initial infection, HSV-1 spreads and infects adjacent sensory neurons in the ganglia, where it enters a latent state. From there, HSV-1 can be reactivated upon cellular stress factors, such as UV-light [83]. Less common, HSV-1 infections lead to encephalitis, meningitis, or keratitis, in particular in immune-compromised patients [84,85,86].

HSV-1 enters cells either upon direct fusion at the cell membrane or via endocytosis (reviewed in [87,88]). Independent of the entry pathway, HSV-1 attaches via the viral glycoprotein gC to heparan sulfate chains on the cell surface [89]. Binding of gC to heparan sulfate is not strictly essential for infection since gC-deficient virus can still enter cells, although about 10-times less efficiently. Another glycoprotein, gB, was found to bind heparan sulfate and thereby represents an additional way of attachment [90]. The initial attachment is further stabilized by binding of gD to one of several different cellular receptors collectively referred to as herpesvirus entry mediator proteins (Hves) [91,92]. Fusion of the viral envelope with the cellular membrane or endosomal membrane requires the glycoproteins gB, gD, and the gH/ gL heterodimer (reviewed in [93]).

After fusion, the viral capsid and tegument proteins are released into the cytoplasm. The capsid, together with some tegument proteins (e.g., UL14, US3), are transported to the nucleus in a dynein-dependent manner along microtubules [94,95]. Empty viral capsids have been observed to accumulate at nuclear membranes and were found to interact with the nuclear pore complex (NPC) [94].

Viral DNA is ejected from the capsid at the NPC, likely through the HSV-1 portal vertex, into the nucleus, and IE-gene expression is initiated by the tegument protein VP16 together with cellular proteins (Oct-1, TFII-B, and TFII-D) [96,97,98]. Loss of VP16, due to a long retrograde transport in axons, has been suggested to be responsible for the transition to a latent state in neurons [99]. IE gene expression, specifically ICP0, ICP4, and ICP27, subsequently triggers transcription of E genes, which encode proteins for viral DNA replication [77]. HSV-1 DNA replication takes place in distinct membrane-less structures called viral replication compartments (VRCs) [100]. Within those VRCs, distinct viral and cellular proteins accumulate and mediate an optimal environment for viral transcription, DNA replication, and capsid assembly and packaging [101].

The seven viral proteins UL9, ICP8, UL5/UL8/UL52 (HP complex), as well as UL30/UL42 (DNA polymerase), are essential for DNA replication (reviewed in [102]). HSV-1 has three origins of DNA replication: two *oriS*, which are located in the *U_S_* repeat regions and one *oriL* within the *U_L_* region [103,104]. UL9, the HSV-1 origin binding protein (OBP), binds and unwinds dsDNA at the replication origins, enabling binding of the viral ssDNA binding protein ICP8 [105,106]. ICP8 was found to directly bind to UL9 and enhance its helicase as well as ATPase activity [107]. ICP8 binds to and stabilizes ssDNA in a sequence-unspecific manner [108]. In addition, UL9 interacts with other viral proteins such as UL8 from the helicase/primase complex (HP complex; UL5/UL8/UL52) as well as the DNA polymerase accessory protein UL42 [109,110]. After an initial unwinding of the replication origin, the HP complex is recruited, which mediates further unwinding by its helicase UL5 and priming through its primase UL52 [111,112]. The HSV-1 genome is replicated by the viral DNA polymerase holoenzyme UL30/ UL42. Packaging of HSV-1 DNA was found to take place only if longer-than-unit-length concatemers are present [113]. This means that the viral genomes must be concatemerized prior to packaging. Early reports hypothesized that genome circularization takes place prior to DNA replication, thus enabling a rolling circle amplification, which inherently generates concatemers [114]. More recent work suggests that concatemers are formed by direct recombination mediated by ICP8 and UL12 [115,116,117].

Capsid assembly requires transcription of viral late genes, including the three structural proteins VP5, VP19C, VP23, and one scaffolding protein (e.g., pre-VP22a or VP21), but does not require any cellular proteins [118,119,120].

Viral DNA is taken up into preformed capsids in an ATP-dependent manner through the portal vertex, which consists of UL6 [121,122]. How HSV-1 acquires its outer layers is still under debate [123,124]. It is widely accepted that mature capsids bud through the inner nuclear membrane, which was shown to be dependent on the cellular endosomal sorting complex required for transport-III (ESCRT-III) [125,126]. The subsequent steps, on the other hand, are still unclear, but two main hypotheses exist. The re-envelopment model suggests that enveloped particles from the perinuclear space fuse with the outer nuclear membrane, thereby losing their first envelope and releasing capsids into the cytoplasm. The final envelope is then gained through budding into Golgi-derived vesicles or Golgi compartments [125,127,128]. A second model suggests that the enveloped capsids travel to the Golgi compartment through the lumen within the endoplasmic reticulum or in vesicles and are released by a secretory route [129,130]. It was further proposed that capsids exit the nucleus via the nuclear pore complex (NPC) and then bud into the Golgi compartment. Although NPCs limit the transport of particles to a maximum size of 39 nm and thereby would not allow HSV-1 capsids with a size of 125 nm to pass, infected cells were shown to have enlarged NPCs [129,131].

#### 2.1.2. HSV-1 Helper Functions

HSV-1 promotes two essential mechanisms necessary for reactivation of AAV replication after a latent infection: AAV Rep expression and AAV DNA replication (see Figure 1 and Table 1). Addition of the HSV-1 helicase–primase complex (HP; UL5/ UL8/ UL52) and the single-strand DNA binding protein ICP8 (gene *UL29*) is sufficient to restore AAV progeny production in a transient AAV-infection model [132].

ICP8 cooperatively binds ssDNA with high affinity in a sequence-independent manner [108]. During HSV-1 infection, ICP8 binds to the unwound ssDNA and to the origin-binding protein UL9. The HP complex is then recruited along with HSV-1 DNA polymerase UL30/UL42, which initiates HSV-1-DNA replication [77,107]. In the context of an AAV/ HSV-1 coinfection, Rep68/78 was found to colocalize with ICP8 as early as 8 h postinfection (hpi) in an AAV ssDNA-dependent manner [101,133,134]. ICP8 directly binds to Rep78. This binding was enhanced in the presence of ssDNA but not dsDNA. Rep78-ICP8 binding was further stabilized in the presence of AAV-ITR sequences, likely due to the binding of Rep78 to the Rep-binding site (RBS) within the ITR sequence. In this context, Rep78 might take on the role of UL9 as the origin binding protein. The role of the HP complex (UL8/UL5/UL52) during AAV DNA replication was analyzed by Slanina et al. [135]. Although UL52 was found to be required for AAV genome replication, its primase activity was dispensable, suggesting it plays a role as a structural protein rather than being enzymatically involved. UL5 helicase activity, on the other hand, was found to be necessary for fully efficient AAV replication. This observation is somewhat surprising as Rep78 and Rep68 both possess helicase domains themselves [67,136]. Although UL5 helicase activity enhanced AAV replication but was not found to be essential, a structural role for UL5 (i.e., the formation of VRCs) during replication could be suggested.

Although transfection of the four HSV-1 proteins (UL5/ UL8/ UL52 and ICP8) is sufficient to restore AAV DNA replication, replication efficiency was less than 1% of what has been observed in the presence of all HSV-1 genes [135]. Thus, other HSV-1 factors are required to fully activate AAV replication.

Rep-proteins play an essential role during the AAV life cycle. In a transient AAV infection, only low levels of Rep expression can be detected in the absence of a helper virus [137,138]. In a latent infection model where the AAV genome is integrated into the host chromosome (e.g., in the AAVtCR cell line), Rep expression must be induced before AAV genome replication can take place. In AAVtCR cells, transfection of a plasmid encoding the HP complex along with ICP8 was not sufficient to initiate AAV replication, presumably due to low levels of Rep-proteins [139]. Transfection of AAVtCR cells with an ICP0-encoding plasmid induced Rep-expression, although to a lower level than that observed upon HSV-1 infection. The immediate-early protein ICP0 is an E3 ubiquitin ligase and a well-known transactivator of HSV-1 gene expression [140,141,142,143]. ICP0 was proposed to activate transcription of AAV *rep* genes in an indirect manner by mediating the protease-dependent degradation of cellular proteins, which inhibit Rep expression [144,145]. Furthermore, the viral transcription factor ICP4 was found to promote Rep-expression, although to a lower level than ICP0 [139]. ICP0/ ICP4 coexpression had a synergistic effect on Rep expression. The addition of another viral transcription factor, ICP22, alone did not result in Rep-expression, but it further increased the level of Rep proteins if expressed together with ICP0 and ICP4. Interestingly, the addition of the viral RNA-processing factor ICP27 during an ICP0/ ICP4 cotransfection decreased the level of Rep proteins. Nevertheless, the addition of those transactivating proteins failed to induce AAV genome replication. Cotransfection of AAVtCR cells with plasmids encoding ICP0, ICP4, and ICP22, in addition to the previously identified HSV-1 helper factors (HP complex and ICP8), led to the detection of replicative forms of the AAV genome. Nevertheless, the presence of those seven HSV-1 proteins was not sufficient to restore AAV replication to levels found during HSV-1 coinfections. A role for the HSV-1 DNA polymerase (UL30/UL42) during AAV DNA replication was suggested, as the addition of phosphonoacetic acid (PAA) during an AAV/HSV-1 coinfection lowered the replication efficiency of AAV [146]. PAA specifically inhibits HSV-1 polymerase, presumably by interfering with its DNA-elongation step [147]. Indeed, it was shown that in suspension, AAV DNA was replicated by the HSV-1 DNA polymerase complex [148]. This observation stands in contrast to observations during AdV coinfection studies where the cellular polymerase is responsible for replicating AAV genomes. Only the cotransfection of plasmids encoding the three HSV-1 trans-activators ICP0, ICP4, and ICP22, HSV-1 DNA polymerase (UL30/ UL42), single-strand binding protein (ICP8), as well as the HP complex (UL5/ UL8/ UL52) are present in AAVtCR cells; all four Rep-proteins were expressed and AAV DNA replicated to levels comparable to those obtained upon HSV-1 coinfection [139].

In a Rep pull-down approach, ICP8, ICP4, UL30, and UL42 were found to directly interact with Rep-proteins at 20 hpi, whereas other helper factors were not identified [134]. Those other helper factors (ICP0 and HP complex) might only be relevant at early timepoints during AAV replication or they might not interact directly with Rep-complexes, as proposed to be the case for ICP0. UL12, on the other hand, was identified to interact with Rep-proteins and localize to AAV VRCs. UL12 is a 5′-to-3′ exonuclease, which, in complex with ICP8, mediates DNA strand exchange and recombination [115,149]. Although apparently not directly contributing to AAV genome replication efficiency, UL12 had an effect on the appearance of different AAV DNA replication forms [134]. In the absence of UL12, the normally observed pattern of the monomeric replication form (mRF) and the dimeric RF (dRF) was less distinct in Southern blot assays, and a more pronounced smear was observed, which indicates the presence of different replication intermediates. It was shown that the exonuclease activity of UL12 was necessary to restore the distinct pattern. Further analysis revealed that the addition of UL12 increases the number of rAAV particles in a vector production setup, indicating that UL12 might support the formation of AAV genome structures suitable for packaging.

In conclusion, the HSV-1 HP complex, in addition to the ssDNA binding protein ICP8, is sufficient to induce AAV replication in a transient infection. During latent infections, Rep-expression needs to be induced through the addition of ICP0 or ICP4, which can be further supported by ICP22. The HSV-1 polymerase further supports AAV replication. The role of UL12 during AAV replication is, to date, not fully understood, but there are indications that it is an additional replication factor supporting the production of infectious AAV particles.

Although most studies have focused on HSV-1 and AAV2 coinfections, it is likely that the same HSV-1 factors also support the replication of other AAV serotypes. A study by Stutika et al. analyzed HSV-1 helper factors supporting AAV5 replication and concluded that the previously revealed helper functions for AAV2 also apply to AAV5 [150]. Since AAV5 is the most distantly related strain of AAV and was, in contrast to AAV1–4, directly isolated from human tissue, it is likely that the same HSV-1 factors, which support AAV2 and AAV5 replication, also support other AAV serotypes [151,152].

In addition to identifying which HSV-1 genes support AAV replication, factors involved in AAV VRC formation were analyzed. VRCs represent an environment that is favorable to virus progeny production by concentrating essential viral, but also cellular, proteins that are necessary for genome replication, capsid formation, and packaging, and prevent the interference of cellular defense mechanisms. Interestingly, in cells coinfected with rAAV2 and recombinant HSV-1 (rHSV-1), distinct, separate VRCs for each virus are formed [101]. Within those distinct compartments, only viral DNA of the respective virus is detected. Rep-proteins were found to localize to AAV VRCs but not HSV-1 VRCs. ICP4, a transcriptional regulator of HSV-1, on the other hand, localized to HSV-1 but not AAV2 VRCs. Interestingly, the HSV-1 ssDNA binding protein ICP8 was recruited to both AAV as well as HSV VRCs, although showing different localization patterns. ICP8 staining within AAV2 VRCs was found to be homogeneously distributed, whereas in HSV-1 VRCs, ICP8 formed a punctate pattern. Those studies also revealed that AAV inhibits HSV-1 replication in coinfected cells (see section below for further details). Colocalization of AAV Rep-proteins and HSV-1 ICP8 was also found in wildtype virus-infected cells as well as in studies where the AAV and HSV-1 genomes were provided by plasmid transfection [133,135]. In contrast, another study using a FISH-approach found that in latently infected AAVtCR cells, wtHSV-1 infection led to the formation of overlapping AAV/HSV VRCs [134]. These studies analyzed AAV and HSV VRCs at different timepoints, where separate VRCs were found at 12–16 hpi and partially or completely overlapping VRCs at 20–24 hpi. This suggests that at early timepoints, distinct VRCs form, which may come together and fuse over time.

### 2.2. Adenoviruses

Among all the viruses that are known, helper viruses for AAV, AdVs, and herpesviruses are the most studied and cited to date [153,154]. Therefore, the molecular mechanisms and the associated viral factors that are involved in driving AAV replication are well-known and described in great detail (see Figure 1 and Table 1). The fact that AdVs are efficient helper viruses for AAV became clear very early. Essentially, the discovery of AAVs as contaminants of AdV preparations almost sixty years ago was one of the first hints that AAV replication may be linked to AdVs in some way [1,2]. However, the first AdV helper factor was identified only ten years later, when Shimojo and his team in 1977 found that AdV12 transformed cells allow the efficient replication of AAV [155]. In this study, they identified the AdV12 large T-antigen, known today as the E1B proteins, as a potential driver of AAV replication. To date, a set of additional AdV helper factors, such as E1B55K, E2A, E4orf6, and the virus-associated RNA (VA RNA), is known to be required in order to induce an efficient AAV replication. In the following two sections, we briefly summarize the importance of these factors in AdV virus biology and their impact on AAV replication.

#### 2.2.1. Adenovirus Biology

The family of the *Adenoviridae* comprises five genera, among which the genus *Mastadenovirus* includes the human AdV species A-G (hAdVA-G). To date, approximately 57 hAdV types are known, and most of them are associated with a pathological disorder such as respiratory diseases, conjunctivitis, or gastroenteritis (reviewed in [156]). The AdV virion is a nonenveloped particle that encapsidates a linear, nonsegmented double-stranded DNA genome of about 35–36 kbs [157]. The common AdV genome encodes roughly 40 proteins that are expressed in a tightly regulated manner and are divided into an early (E) and a late (L) phase, both taking place in VRCs within the nucleus of the infected cell [157,158,159]. The first early protein that is expressed in an AdV infected cell is E1A, a universal viral transcription regulator that acts together with multiple cellular factors in order to orchestrate viral transcription and modulate the cellular antiviral host response and apoptosis. E1A stabilizes the preinitiation complex (PIC) and it recruits cofactors that stabilize the expression of distinct viral and cellular genes [66,157,160,161]. An important aspect of the AdV E1A transcriptional regulation is its interaction with cellular factors of the cAMP/PKA signaling pathway. This has a relevant implication during the AAV Rep-mediated inhibition of AdV replication, which we discuss later in this review. The cAMP/PKA pathway regulates a set of cellular functions, such as transcription, proliferation, and differentiation (reviewed in [162]). The cAMP-dependent transcription is controlled by cAMP-responsive elements (CREs) located at a set of distinct cellular promoters that can bind the CRE-binding protein (CREB), which is phosphorylated by the protein kinase A (PKA) upon different stimuli in the cell, such as increased cAMP levels [163]. The phosphorylated CREB protein then recruits the CREB-binding protein (CBP) to the CRE promoters, inducing the formation of the PIC and the initiation of transcription [164]. In fact, all but one AdV gene promoter comprise CRE elements and hence are directly controlled by this cellular signaling pathway. In addition, the viral E1A protein can directly interact with two cellular factors that are involved in this pathway, CREB and ATF-1, both CRE binding proteins, and hence has a direct impact on the regulatory functions of these proteins [165]. Importantly, the AdV E2 gene complex that encodes for proteins essential for viral DNA replication is directly controlled by E1A and the cAMP/PKA pathway [165,166,167]. This has been confirmed by studies demonstrating that E2 expression is directly activated by PKA, and it is inhibited by the PKA-specific inhibitor protein (PKI) and CREB-A, a cellular antagonist of CREB [167]. Three major AdV proteins are encoded by the *E2* gene complex, the precursor terminal protein (pTP), the AdV DNA polymerase (AdVpol), and the DNA-binding protein (DBP). These proteins constitute the main DNA replication complex of AdV and, upon expression, are recruited together with a set of cellular factors to the AdV origin of DNA replication that is located at the inverted terminal repeat (ITR) of the genome (reviewed in [159,168]). Other relevant early AdV proteins belong to the *E1B* gene complex that encodes for the E1B19K and E1B55K oncoproteins. The E1B19K protein inhibits the E1A-induced apoptosis in the cell and triggers autophagy via the interaction with Beclin-1 [169,170,171,172]. The E1B55K protein, on the other hand, binds and inhibits the tumor suppressor protein p53, and therefore promotes cell-cycle progression and inhibits apoptosis [173,174,175]. The tightly regulated interplay between the AdV early proteins E1A and E1B represents the core of the AdV oncogenic nature and has been studied intensively (reviewed in [173,176,177]). The last important AdV early protein we will discuss here is encoded by *E4orf6*. The genomic *E4* region is subdivided into several ORFs and encodes for seven predicted mRNAs, of which six have been detected in infected cells (reviewed in [178]). Not all ORFs from the *E4* region are essential for the virus to grow in cell culture. In fact, only E4orf3 and E4orf6, which have redundant functions, are required in order to complete a lytic life cycle [179,180]. E4orf6 is a versatile protein that forms a functional complex with E1B55K and is involved in a variety of viral functions, such as DNA replication, RNA processing, or shut-off of host cell protein synthesis [178,181]. For example, the E4orf6/E1B55K complex stabilizes the viral genome during replication and prevents it from unwanted concatemerization [182]. In addition, it inhibits a cellular DNA damage response to double-strand breaks by targeting the Mre11 protein for proteasomal degradation and hence disrupts the Mre11/Rad50/NBS1 (MRN) complex which is an important mediator of the cellular DNA-damage response pathway [183]. Lastly, we will briefly discuss the impact of the virus-associated RNA (VA RNA) during the AdV replication cycle. All AdV types express relatively high numbers of noncoding viral RNA transcripts throughout the entire replication cycle, and they can be detected starting at 18 hpi [184,185]. At least two highly structured VA RNAs (VA RNA_I/II_) are expressed by all human AdV types and constitute essential regulatory molecules during infection. Similar to cellular noncoding RNAs, VA RNAs interact with a variety of cellular proteins, and hence they are involved in regulating many antiviral and innate immune response pathways. The best-studied and, therefore, well-understood function of AdV VA RNAs is the inhibition of the cellular innate immune protein double-stranded (ds)RNA-activated kinase (PKR) [185,186]. By inhibiting PKR, the infected cell can no longer induce the PKR-mediated shut-down of general translation, ensuring efficient virus protein synthesis during infection [187]. Importantly, the VA RNA-mediated inhibition of PKR, therefore, confers to the well-known interferon resistance of AdVs.

#### 2.2.2. AdV Helper Functions

The most commonly used, and hence best-studied helper functions originate from the human AdV type 5 (hAdV5). However, many studies were conducted using other AdV types such as hAdV2, and therefore, we generally refer to AdVs in the next sections. The distinct AdV helper functions for AAVs are well known and have been studied in great detail [153,154,188,189]. AdV helper genes have successfully been utilized for AAV vector production in the past (see Section 4). The minimal set of AdV helper factors for efficient AAV replication and hence, the production of AAV progeny, consists of five AdV molecules: E1A, E1B55K, E2A, E4orf6, and the VA RNA (see Figure 1 and Table 1). Here, we will discuss the impact of each helper factor during the life cycle of AAV and explain the molecular mechanisms underlying these AdV helper functions.

*E1A*. AdV E1A and E2A proteins represent the most important helper factors because they activate the AAV *p5* and *p19* promoters that are controlling the expression of AAV Rep proteins [190,191,192,193]. The E1A-induced *p5* activity has been found to be key during AAV replication by two independent studies almost 40 years ago [194,195]. Tratschin et al. found that AAV titers were dramatically increased when the AdV E1A protein was transiently overexpressed in AAV-producing cells. In addition, they found that AAV titers were also increased in Hek293 cells, a cell line stably expressing E1A, when compared to HeLa cells that do not express E1A [194]. The study of Richardson and colleagues found that AAV gene expression, in general, was increased in AAV-infected cells when AdV mRNAs were introduced by microinjection [195]. Importantly, it has been found that the *p5* promoter contains an E1A-inducible element that is employed by E1A and is believed to be the core element for the E1A-driven *p5* activity [192]. Lastly, E1A can interact with YY1 and relieves the YY1-mediated repression of the *p5* promoter [192,196].

*E1B55K*. This subunit functions together with E4orf6 as a helper factor complex for AAV. This protein complex promotes AAV second-strand synthesis and viral DNA replication [197,198]. Another important aspect of the E1B55K helper function is the facilitated export of AAV mRNA and the coinciding inhibition of cellular mRNA export, both promoting AAV gene expression [199]. Although the exact molecular mechanisms are not fully understood, it has become clear that the E1B55/E4orf6 protein complex constitutes an essential AAV helper factor, especially for AAV vector production.

*E2A*. This single-stranded DNA binding protein (ssDBP) can stimulate AAV DNA replication in vitro and is involved in the splicing of Rep proteins [200,201]. Moreover, AAV Rep proteins can interact with ssDBPs such as E2A, recruit them to AAV VRCs, and enhance viral replication [202]. E2A is involved in a variety of additional functions that facilitate AAV replication, such as mRNA processing and export or capsid production [200,203,204].

*E4orf6*. As described above, E4orf6 can form a complex with E1B55K that is involved in facilitating AAV mRNA export from the nucleus [199]. However, E4orf6 also promotes AAV replication by enhancing second-strand synthesis [198,205]. Another important helper function of E4orf6 might be the abovementioned degradation of Mre11 that is part of the MRN complex. Evidently, it has been shown that the MRN complex limits AAV transduction and replication, which represents a major problem, especially during AAV vector production [206,207]. An interesting observation was made by Allen et al., who found that E4orf6 constitutes the only AdV helper factor necessary if AAV Rep and Cap proteins are overexpressed in AAV vector producing cells [208]. This suggests that Rep-, together with Cap proteins, can somehow compensate for all other AdV helper factors, giving the possibility to greatly improve AAV vector production protocols. However, E4orf6 also has negative effects on AAV replication. For example, it mediates the degradation of *de novo*-assembled AAV capsids and Rep52 in a ubiquitin-dependent manner [189]. This hurdle, however, can be overcome in coinfected cells by fine-tuning the expression profile of AdV genes that is a hallmark of the AAV-mediated inhibition of AdV replication; this is discussed below.

*VA RNA*. It was proposed that the helper function of the AdV VA RNA is mainly based on the VA RNA-mediated degradation of PKR [209]. In fact, a small element in AAV *p41*-generated RNAs naturally induces PKR in the infected cell and promotes the PKR-driven phosphorylation of eIF2alpha that has a negative effect on AAV protein synthesis. Another critical helper function of the AdV VA RNA_I_ is the cooperative enhancement of the AAV Cap protein expression [210]. In particular, the AdV VA RNA_I_, together with AdV DNA-binding proteins, are required for efficient synthesis of AAV structural proteins and hence are important helper factors that are involved in AAV Cap expression and assembly.

Of note, two additional AdV factors have been associated with AAV replication: protein IX and E1B19K. However, these factors are not required for AAV replication; rather, they are known to enhance AAV vector titers when coexpressed with the essential AdV helper factors E1A, E1B55K, E2A, E4orf6, and VA RNA [211,212]. Another AdV helper function that may have a significant impact on the AAV replication cycle is the recruitment of AAV genomes into AdV VRCs. Similar to what was proposed for HSV-1, Weitzman et al. described that both wildtype and recombinant AAV genomes colocalize with AdV2 VRCs [213]. Recruitment of AAV genomes into helper virus VRCs may be seen as an attempt of AAV to take advantage of the molecular environment within helper virus VRCs that promotes AAV transcription and genome replication.

### 2.3. Other Helper Viruses

#### 2.3.1. Other Herpesviruses

As mentioned above, several other herpesviruses can provide helper functions for AAV, such as HCMV, HSV-2, VZV, or HHV-6 [9,72,73,74]. HSV-2 and VZV belong to the subfamily *Alphaherpesvirinae*, the same as HSV-1, whereas HCMV and HHV-6 belong to the subfamily *Betaherpesvirinae*. Since most herpesviruses from a certain subfamily share genetic and structural similarities, it is tempting to speculate that herpesviruses belonging to the same subfamily may provide the same helper functions for AAV. Indeed, very early studies on HSV-1 and HSV-2 revealed that the production of AAV preparations in cells coinfected with either helper virus was equally efficient, most probably because the helper functions of these two herpesviruses are very similar [9,72,73]. Efficient AAV replication was also observed in cells that were coinfected with HCMV [9]. However, the exact HCMV helper factors have not been investigated further and hence remain to be identified. HHV-6 helper functions are associated with the HHV-6 Rep protein, a homolog of the AAV Rep68/78 proteins, which can efficiently enhance AAV replication, most probably by mimicking the molecular functions of AAV Rep proteins [74]. Interestingly, it has been proposed that the HHV-6 Rep homolog was acquired from AAV as a result of the natural transfer of genetic information during the coevolution of these two viruses [214].

#### 2.3.2. Papillomaviruses

The role of papillomaviruses, especially HPV-16, in AAV biology gained great interest in the past years after it was found that both HPV-16 and AAV can infect and reside in cervical cancer tissue and that AAV reduces the risk of developing HPV-16-induced cervical cancer [10,215,216,217,218]. A number of studies have addressed the molecular interaction between HPV-16 and AAV in coinfected cells, resulting in the identification of HPV-16 helper functions for AAV replication [219,220,221,222,223]. However, it turned out that HPV-16 may not be a complete helper virus for AAV, as AAV does not replicate very efficiently when solely HPV-16 helper factors are present. For example, the HPV-16 E1, E2, and E6 proteins can greatly enhance rAAV and wtAAV production, but only when the full set of AdV helper factors is present [220]. Similarly, You et al. found that the same set of HPV-16 proteins can enhance AAV replication in epithelial raft cultures [222]. The HPV-16 helper factor E1 has recently been investigated in more detail, and it has been found that its helper effect can be attributed specifically to the E1 carboxyl domain [221]. In vivo and in vitro experiments have revealed that the AAV Rep and the HPV-16 E1 proteins can directly interact and modulate the Rep ATPase activity [224,225]. Despite the fact that HPV-16 E7 is believed to have minimal effect promoting AAV replication, the molecular details underlying the E7-driven boost in AAV replication is not well understood [222]. Actually, it has been seen that the E7 protein even has an inhibitory effect on AAV replication, possibly because of the repression of AAV *cap* expression [222].

#### 2.3.3. Bocaviruses

The human bocavirus 1 (HBoV1) is a small autonomous parvovirus that has been described very recently as a novel helper virus for AAV [12]. Wang et al. identified a small set of HBoV1 helper factors that can efficiently rescue AAV replication. In particular, the NP1 and NS4 proteins, together with the viral long noncoding RNA (BocaSR), constitute a minimal set of helper factors that can trigger the replication of a duplex AAV genome in transfected HEK293 cells. Interestingly, in AAV-infected cells, the minimal set of HBoV1 helper factors comprise NP1, BocaSR, and NS2, but not NS4 [12]. This interesting observation led to a couple of assumptions on the molecular functions of NS2 and NS4; however, the detailed molecular mechanism of how HBoV1 supports AAV replication is not known. The same research group then utilized this bocavirus helper system in order to develop a novel rAAV vector production platform that may have implications in future AAV gene therapy vector production [226]. They combined a distinct set of AdV and bocavirus helper genes and found that rAAV titers were significantly increased when compared to rAAV vector titers that have been produced with either AdV or bocavirus helper factors alone. Lastly, other bocaviruses, such as HBoV2–4 and the gorilla bocavirus, were recently found to provide helper factors for the generation of AAV gene therapy vectors (GBoV) [227].

## 3. AAV-Mediated Inhibition of Helper Virus Replication

Viral coinfections inevitably lead to competition for cellular resources. In terms of AAV/helper virus coinfections, competition also exists over helper factors. Although AAV has a very limited number of viral proteins, it has still managed to evolve a variety of strategies to suppress helper virus replication.

### 3.1. AAV Inhibits HSV-1 DNA Replication

AAV serotypes 2, 3, and 5 were found to inhibit HSV-1 DNA replication [228]. During an AAV/HSV-1 coinfection, HSV-1 replication is suppressed to ensure the availability of cellular resources, but at the same time, HSV-1 helper factors are synthesized. Since all HSV-1 helper factors belong to the group of immediate-early or early genes, it is not surprising that AAV has evolved to specifically suppress HSV-1 DNA replication, which disables transcription of late genes (see sections above for further information about HSV-1 biology and helper factors) [229]. It has further been shown that the presence of AAV Rep68 or Rep78 protein is necessary and sufficient to inhibit HSV-1 replication [229,230]. Although Rep endonuclease activity was found to be dispensable, its ATPase/ helicase activity and the DNA binding domain were essential. These findings suggest that Rep-proteins can directly bind to and interact with HSV-1 DNA [231]. It has indeed been shown that Rep68 binds to nine consensus sequences, harboring the minimal Rep-binding site (RBS) motif GAGYGAGC, on HSV-1 DNA and inhibits replication. Further, Rep68 was shown to inhibit replication of any DNA template when binding is facilitated.

During HSV-1/AAV coinfection in cell culture, both viruses replicate, indicating that AAV-mediated inhibition of HSV-1 is not complete. This raises the question of how those different viruses can coexist in a cell population. AAV and HSV-1 replicate during different cell cycle phases and hence replicate in two different ecological niches [232]. In the absence of AAV, HSV-1 replication is cell-cycle independent and blocks cell cycle progression in the G1 and G2 phases [233,234,235,236]. AAV gene expression and replication were found to preferentially occur in S/G2 phases of the cell cycle [232]. Restriction of AAV replication and gene expression to S/G2 was not dependent on second-strand synthesis as self-complementary rAAV showed the same cell cycle preference. During AAV2 coinfection, HSV-1 replication becomes restricted to G1 in an AAV Rep-protein-dependent manner.

Furthermore, AAV was shown to delay HSV-1 mediated degradation of DNA PKcs [237]. AAV-mediated modification of the cellular DNA damage response (DDR) induced by HSV-1 points to an additional, indirect mechanism used by AAV to suppress HSV-1 replication and create an environment in favor of its own replication.

### 3.2. AAV2 Inhibits AdV Replication

Inhibition of helper virus replication that is mediated by AAV has a significant impact on the AAV life cycle. The competition for cellular resources and compensation of the viral burden in coinfected cells is reduced and favors the AAV replication cycle. However, minimizing helper virus replication can also reduce the availability of viral helper factors. In order to guarantee both the inhibition of the helper virus replication and the availability of required helper factors, AAV has established elegant molecular strategies. One such example is the inhibition of AdVs. AAV can inhibit AdV replication in coinfected cells in two ways: a direct and an indirect mechanism, both mediated by AAV Rep proteins. Direct inhibition of AdV replication involves the suppression of AdV gene expression. The AAV Rep78 protein can bind to two promoter sequences on the AdV genome, E2A [238], and the major late transcription promoter (MLP) [239]. Binding to both promoter sequences occurs independently of a consensus Rep-binding site (RBS). As for the MLP, Rep-binding occurs at a well-defined 55-bp fragment and is mediated via interaction with the cellular TATA box-binding protein (TBP) [239]. Association of Rep to these two promoters results in the inhibition of AdV early and late gene expression and reduces AdV replication to some extent. A more detailed AdV gene expression profile in AdV/AAV coinfected cells revealed that the Rep-mediated inhibitory effect on AdV transcription did not affect all genes equally [240]. In particular, the expression of E4 and the late genes was strongly inhibited, whereas E1A and E2A were not, or just to a lesser extent. Inhibition of E4, however, stands in contrast to the fact that E4orf6 is an important helper factor required for the degradation of the MRN complex that naturally inhibits AAV replication [206,207]. Furthermore, in the same study, it was hypothesized that the AAV-mediated inhibition of AdV replication may also occur via direct interference with the AdV DNA replication process. In fact, it is tempting to speculate that a mechanism similar to the inhibition of HSV-1 DNA replication can inhibit AdV DNA replication as well. The AAV Rep68/78 DNA-binding domain (DBD), together with the functional helicase domain, are required to bind and unwind double-stranded DNA templates and induce a DNA damage response (DDR) that is not compatible with any DNA replication process [229,231,241]. This approach constitutes a potential strategy to inhibit any helper virus if Rep can bind the corresponding genome. In addition, AAV has evolved indirect mechanisms to inhibit AdV replication. More precisely, AAV Rep78 proteins can restrict AdV replication via inhibition of PKA [242]. As mentioned above, AdVs utilize the cAMP/PKA signaling pathway to induce the switch from early to late replication stage by regulating transcription that is controlled by E1A [166,167]. AAV Rep52/78 proteins both comprise C-terminal PKA-specific inhibitor protein (PKI)-like motifs that directly interact with PKA and inhibit the cAMP/PKA signaling pathway and thus the AdV late gene expression [242]. Lastly, a short cis-regulatory element from the 3′-end of the *rep* gene, termed the Rep inhibition sequence for adenoviral replication (RISAd), has been identified to inhibit AdV replication [243]. This mechanism requires a functional AAV *p40* promoter that gives rise to short *p40*-associated transcripts in AdV vectors, which comprise the RISAd. The authors of this study hypothesized that the RISAd sequence leads to the stalling of the RNA polymerase II (RNApolII) during transcription that may interfere with the AdV DNA polymerase during DNA replication. This AAV-mediated inhibitory effect on AdV DNA replication has an impact on the design of novel AdV/AAV hybrid vectors for gene therapy.

### 3.3. AAV Inhibits Other Helper Viruses

AAV has developed a variety of molecular strategies to inhibit helper virus replication without preventing essential helper functions. The inhibitory effects of AAV Rep proteins on AdV and HSV have been studied extensively, and hence, the underlying mechanisms are well known (see Section 3.1 and Section 3.2). However, the inhibition of other helper viruses has not been investigated and is poorly understood. One exception is the interaction of AAV with HPV-16. The interplay between these two viruses on a molecular basis has gained great interest in the past years as it was proposed that AAV may inhibit HPV-16 in coinfected cells and therefore reduce the risk of HPV-16 induced cervical carcinomas [215,217,218]. This notion has driven the investigation of a number of studies that have particularly addressed the question of whether or not AAV may inhibit HPV-16 and, if it does, what the molecular mechanisms behind this phenomenon are. In fact, it has been found that AAV Rep proteins can suppress the papillomavirus promoter and hence inhibit the transformation of papillomavirus-infected cells in cellulo and in vivo [244,245,246]. Unfortunately, the hype around HPV-16 and AAV eased when new studies on the incidents of cervical cancer in the context of AAV seroprevalence showed that there is no correlation between AAV seroprevalence and the occurrence of HPV-16-induced carcinomas [247]. Interestingly, almost all AAV-mediated helper virus inhibition mechanisms rely on AAV Rep proteins that directly or indirectly interfere with a variety of functions that drive helper virus replication. The potential of AAV Rep proteins to inhibit helper viruses has led to the hypothesis that Rep may also inhibit viruses other than helper viruses. Indeed, a study from the Carter lab showed that AAV Rep proteins can also inhibit RNA viruses such as the human immunodeficiency virus type 1 (HIV-1), an observation that triggered hopes in the development of potential HIV-1 therapies [248]. However, this phenomenon was not further investigated and remains an interesting observation only.

## 4. AAV Vectors for Gene Therapy

Advances in our understanding of AAV biology have been promoted to a large extent by the recognition that this virus is a particularly well-suited vehicle for gene therapy. Knowledge of AAV biology, in turn, is instrumental to innovative therapeutic vector development.

Among the advantageous features of AAVs as gene therapy vectors is the fact that these viruses are not known to cause any disease. Moreover, AAV can be produced at very large titers, support long-term transgene expression even in the absence of transgene integration into the host genome, and infect a large variety of different cells. By using the capsid proteins of different virus serotypes, both natural (serotypes 1–12) and synthetic serotypes, AAV vectors can be engineered to target, at least to some extent, specific cells and tissues (reviewed in [249]).

The main disadvantages of AAV as a gene transfer vehicle are its low transduction efficiency, which requires high vector doses and its small transgene capacity of approximately 4.5 kb. The latter prevents the exploitation of one key feature of wildtype AAV, the site-preferential integration of its genome into the *AAVS1* site on human chromosome 19. That process requires the AAV large *rep78/68* genes [250,251], which, together with a therapeutic transgene, would, in most cases, exceed the transgene capacity of the vector. To overcome this problem and exploit the site-preferential integration capacity of wtAAV for the genomic integration of therapeutic transgenes, several different hybrid vectors have been developed. Such hybrid vectors are designed by placing the AAV *rep* genes and the ITR-flanked therapeutic transgenes onto the vector genomes derived from other viruses (e.g., AdVs or herpesviruses; see Section 4.2). Another disadvantage of standard rAAV vectors is that transgene expression requires prior second-strand synthesis, which delays gene expression, at least in post-mitotic cells such as neurons. To overcome this issue, self-complementary (sc)AAV vector genomes have been developed that can self-anneal and support gene expression without prior second-strand synthesis [252].

AAV-derived vectors are the most widely used gene transfer vehicles in clinical gene therapy, and three different AAV-based gene therapy products have already been authorized, including Glybera (lipoprotein lipase deficiency), Luxturna (Leber congenital amaurosis 2), and Zolgensma (spinal muscular atrophy). Recombinant AAV vectors are produced by transfecting a plasmid that contains an rAAV genome consisting of the AAV ITRs flanking a transgene cassette with a therapeutic gene of interest, together with a plasmid that provides the AAV *rep* and *cap* genes, as well as helper virus genes into cells. Alternatively, the AAV *rep/cap* genes and helper virus genes can be provided by transfection of more than one plasmid or by infection with helper viruses. In the presence of AAV *rep/cap* and helper virus genes, the plasmid-cloned rAAV genomes are replicated and packaged into AAV capsids. The resulting rAAV vectors can then be purified by a variety of different methods. Different strategies for clinical-grade AAV vector production, which are based on transfection, transfection, and infection, and infection alone, are described in Section 4.1 below.

### 4.1. Production of AAV Gene Therapy Vectors

AAV vectors have conventionally been produced by transfection of a plasmid that contains the rAAV genome with the therapeutic gene of interest and a plasmid that encodes both AAV *rep/cap* and AdV5 helper genes encoding *VA RNA*, *E2a,* and *E4orf6* by calcium phosphate precipitation into human embryonic kidney 293 cells (HEK293), which encode AdV5 helper genes *E1a/b* [193,253]. Transfection protocols with the AAV *rep/cap* genes and the AdV helper genes separated on two different plasmids have been described as well [193,254]. These 2- or 3-plasmid transfection protocols are employed in many different laboratories and vector core facilities, although the vector purification strategies differ widely. Common to all methods that depend on transfection is the lack of scalability, at least with adherent cells. Transfection of cells grown in suspension in bioreactors allows vector production to be scaled up to some extent [255].

AAV vectors can also be produced in cell lines that express AAV *rep/cap* (packaging cells) or both AAV *rep/cap* and the rAAV genome (producer cells). For rAAV production, producer cells are infected with a helper virus [256]; packaging cells are transfected with a plasmid containing the rAAV vector genome and infected with a helper virus. Alternatively, to avoid the transfection step and allow scale-up, packaging cells can be infected first with an *E2b*-deleted AdV to induce *rep/cap* expression and then with an AAV/AdV hybrid virus that contains an rAAV vector genome within the AdV helper genome in place of *E1* [257]. All protocols that use wildtype or recombinant viruses to provide helper functions for rAAV production require efficient purification steps to remove the helper viruses from rAAV vector stocks. Another problem of using packaging cells or producer cells for rAAV production is cell stability and the changing cell properties with increasing passage numbers.

HSV-1 has also been described to provide helper functions for productive AAV replication and has indeed been used as a helper virus for the production of clinical-grade rAAV vector stocks. Specifically, the coinfection of cells with two replication-defective rHSV-1 vectors, one containing the AAV *rep/cap* genes, the other the rAAV vector genome, results in the efficient production of rAAV vectors [258,259,260,261]. The drawbacks of using rHSV-1 vectors for rAAV production include the difficulty of generating rHSV-1 vectors and the requirement to completely remove contaminating helper virus from rAAV vector stocks.

The rAAV gene therapy product Glybera is manufactured using a baculovirus system. Specifically, Sf9 insect cells are infected with three different recombinant baculovirus vectors that contain the AAV *rep* and *cap* genes and an rAAV genome, respectively. Alternatively, the AAV *rep* and *cap* genes have been combined on a single recombinant baculovirus [75,262,263]. The system is scalable and does not require a mammalian helper virus but is limited by the low genetic stability of recombinant baculoviruses [264].

### 4.2. AAV Hybrid Vectors

One interesting property of wtAAV is that it can integrate into the host cell genome, preferentially at a site termed *AAVS1* on human chromosome 19, a capacity that depends on the AAV ITRs and the large AAV Rep68/78 proteins [250,251]. While rAAV vectors contain the ITRs flanking a therapeutic transgene, the *rep* genes are excluded because of the small transgene capacity of AAV and because the genomic insertion of the AAV *rep* gene, which encodes a multifunctional virus protein along with the therapeutic transgene, is not desired. To exploit the site-preferential genomic integration capacity of AAV and to overcome its size restriction, hybrid vectors based on HSV-1 or AdV have been constructed that contain an ITR-flanked rAAV genome and, outside of that cassette, the large AAV *rep* genes [265,266,267,268]. For example, HSV/AAV hybrid vectors have been shown to mediate the insertion of transgene sequences of up to 100 kb into *AAVS1* and support long-term transgene expression [267,269]. However, hybrid vector titers were relatively low because the AAV2 *rep* genes inhibited the HSV-1 replication machinery [101,229,231,267,270]. The combined DNA-binding and ATPase/helicase activities of AAV2 Rep78/68 have opposing effects on HSV-1 and AAV2 replication. While these two domains are essential for AAV2 DNA replication and genomic integration, they contribute to the inhibition of HSV-1 replication. Therefore, the insertion of AAV2 *rep78/68* genes into HSV-1-derived hybrid gene transfer vectors to mediate the genomic integration of AAV2 ITR-flanked transgene cassettes in the target cell appears not to be compatible with the efficient production of hybrid vector stocks. However, the analysis of many different mutant Rep78/68 proteins revealed one protein with a point mutation within the ATPase/helicase domain (D to Y transition at amino acid position 371) that unexpectedly allows the simultaneous replication of both AAV2 and HSV-1 [241] and supports the efficient production of HSV/AAV hybrid vectors.

## 5. Perspectives

Adeno-associated viruses (AAVs) differ from most other viruses as they require not only a host cell for productive replication but also the simultaneous presence of a helper virus in the same cell. Despite its very simple genome, which encodes only a small number of genes, AAV is nevertheless able to successfully compete with its genetically much more sophisticated helper viruses. Thus, the complex relationship between AAV and its helper viruses and the coinfected cell provides a unique model to study the molecular mechanisms of interactions between competing viruses in cells. Understanding these mechanisms is not only of interest in fundamental virology and cell biology but may also have practical relevance and wider implications for biomedical research. For example, rAAV vectors have gained significant importance as gene delivery vehicles in human gene therapy [271,272,273,274], and the oncosuppressive and antiproliferative effects of AAV may have broad applications in cancer therapy.

The basic knowledge gained in AAV research over the past thirty years has been a substantial boost for AAV-based gene therapy, giving rise to a variety of AAV-based gene therapy vectors that are currently in clinical trials or already FDA-approved, such as Glybera, Luxturna, and Zolgensma. However, AAV vector production and delivery, as well as the transgene expression efficiencies, are still not satisfactory and have to be improved in order to make more reliable and safer AAV-based gene therapy vectors. For example, many molecular aspects in AAV biology are still not fully understood, such as AAV trafficking in the cell, the uncoating process, second-strand synthesis, or, most importantly, innate and intrinsic immune responses that AAVs and AAV-based vectors provoke in humans. The latter has been a major concern for many years and is seen as one of the main reasons for poor vector performance in gene therapy applications. Intriguingly, it was found that AAV vectors do not trigger an immune response in mice, but do in humans—the reason is still unclear [275,276]. Given the fact that approximately 70–80% of the world population is seropositive for neutralizing antibodies against one or more types of AAV, it is surprising that many questions about the AAV life cycle in vivo remain to be answered. This includes questions about the route of transmission, the molecular interactions with the host and helper viruses, and, most importantly, about where in the body AAVs replicate or whether AAV genome integration is a process that naturally happens in vivo as well. Especially in the context of HPV-16 infections, these questions have a relevant impact and need to be addressed further in order to clarify the contradictory picture we have at the moment regarding the molecular interactions between AAV, HPV-16, and the host. While the integration process of the AAV genome into the host cell genome has been studied extensively in cellulo, less is known in vivo. This is mainly due to the lack of information about where in the host AAV replicates. While genomic integration of AAV vectors is not of particular interest in gene therapy, understanding the integration process of wtAAV in vivo would still be of interest. So far, no evidence points towards an integration process in vivo; rather, the AAV vector genomes remain in an episomal state and are lost after several rounds of replication [251,277,278,279,280]. Site-specific integration into the *AAVS1* locus in the human genome would require the presence of the large Rep proteins Rep68/78. However, it has been shown that overexpression of these proteins causes cytotoxicity due to a Rep-mediated DDR and the subsequent apoptosis of the cell. Mutant Rep proteins that still maintain the integration activity but lack the capacity to induce cytotoxic side effects may overcome this problem. Another AAV Rep-specific issue is the Rep-mediated inhibition of helper viruses. The small AAV transgene capacity has led to the generation of AAV hybrid vectors that comprise genetic components of either HSV-1 or AdV. However, the production of such hybrid vectors has been challenging, especially in cells that coexpress the Rep68/78 proteins. The development of Rep mutants, such as the Rep68-D371Y, that lack the capacity to inhibit HSV-1 replication but still maintain the relevant functions for AAV replication and integration, may overcome this problem [241]. Another aspect that remains to be improved is rAAV vector infectivity. It has not been possible to produce rAAV vector stocks that display similar infectivity as wtAAV stocks. This is due to the fact that rAAV vector packaging is not as efficient as wtAAV packaging and therefore leads to the accumulation of many empty particles in the vector stock [281]. The reason for this phenomenon and solutions to solve this problem are not known yet. A relevant issue in the past that was partially solved is AAV vector specificity. In particular, the targeted delivery of AAV vectors has not been possible, but in recent years, the development of AAV hybrid vectors that contain hybrid capsids from different AAV serotypes has greatly improved AAV vector specificity. Additional work in this field will give rise to an even greater variety of AAV vector types that can be targeted according to the application they are needed for. Lastly, an often-forgotten aspect of AAV and AAV vector research is the great potential of this virus as a tool to study basic cellular biology.

## Figures and Tables

**Table 1:** Helper virus factors involved in supporting adeno-associated virus (AAV) replication.

Helper Virus	Gene Product	Native Function	Helper Function	Essential For AAV	References
HSV-1	UL5	HP complex helicase subunit, unwinds DNA at replication fork	Promotes AAV genome replication, requires UL5 helicase activity	Yes	[132,135]
	UL8	HP complex subunit, stimulates enzymatic activity of UL5 and UL52	Promotes AAV genome replication	Yes	[132,135]
	UL52	HP complex primase subunit, primase activity during DNA replication	Promotes AAV genome replication	Yes	[132,135]
	ICP8	ssDNA binding protein, required for HSV-1 genome replication	Promotes AAV genome replication, binds Rep78 and AAV ssDNA	Yes	[132,133]
	ICP0	E3 ubiquitin ligase, trans-activator of HSV-1 gene expression	Supports AAV Rep-expression	No	[139]
	ICP4	Major viral transcription factor	Supports AAV Rep-expression	No	[139]
	ICP22	Transcriptional regulator	Supports AAV Rep-expression	No	[139]
	UL30	HSV-1 polymerase catalytic subunit	Enhances AAV genome replication	No	[139,148]
	UL42	HSV-1 polymerase subunit	Enhances AAV genome replication	No	[139,148]
AdV5	E1A	General transcription factor, AdV early promoter activation, oncogene	AAV promoter activation, drives cells to S-phase	Yes	[190,192,194,195,211]
	E1B19K	Inhibits proapoptotic Bcl-2 homologs (Bax and Bak), induces autophagy, oncogene	Enhances AAV vector titers	No	[211,212]
	E1B55K	In complex with E4orf6 it prevents E1A mediated p53 stabilization, oncogene	Involved in AAV mRNA export, promotes AAV second-strand synthesis	Yes	[153,175,197,199,211,212]
	protein IX	Minor component of the AdV capsid, capsid stability	Molecular function unclear, enhances AAV vector titers	No	[211]
	E2A	ssDNA binding protein, viral DNA replication & mRNA processing	AAV promoter regulation, AAV genome replication, Rep splicing, capsid protein production	Yes	[193,200,201,202,203,204,211]
	E4orf6	In complex with E1B55K it prevents E1A mediated p53 stabilization, supports viral DNA replication and RNA processing	Promotes AAV second-strand synthesis, inhibits the MRN complex	Yes	[29,153,193,198,201,205,208]
	VA RNA	inhibits the eIF-2 protein kinase, promotes viral protein translation	Prevents E4orf6/E1B mediated degradation of AAV capsids & Rep52	Yes	[29,189,193,201,210]
HPV-16	E1	HPV-16 DNA replication, binds origin of DNA replication on the HPV-16 genome	Binds AAV Rep-ITR, nicking activity, complements AdV5 helper factors, increases rAAV titers, augments *rep* and *cap* expression	No	[220,221,222,224,225]
	E2	Increases p53 levels, activates transcription, inhibits E6	Enhances AAV titers	No	[10,222]
	E6	Oncogene	Complements AdV5 helper factors, increases rAAV titers, augments *rep* and *cap* expression	No	[219,220,222]
HBoV1	NS2	Trans-activation of viral promoters	Promotes AAV second-strand synthesis, not essential when AAV genome is a duplex	(Yes)	[12,226]
	NS4	Trans-activation of viral promoters	Activation of AAV promoter	(Yes/No) ^1^	[12,226]
	NP1	Processing of viral pre-mRNA, trans-activation of viral promoters	Activation of AAV promoter	Yes	[12,226]
	BocaSR	Regulates NS1, NS2, NS3, NP1 expression and viral DNA replication	Not known	Yes	[12,226]

Essentiality is unclear ^1^.
